# Recent Trends in Graphene/Polymer Nanocomposites for Sensing Devices: Synthesis and Applications in Environmental and Human Health Monitoring

**DOI:** 10.3390/polym14051030

**Published:** 2022-03-04

**Authors:** Elisa Toto, Susanna Laurenzi, Maria Gabriella Santonicola

**Affiliations:** 1 Department of Chemical Engineering Materials Environment, Sapienza University of Rome, Via del Castro Laurenziano 7, 00161 Rome, Italy; elisa.toto@uniroma1.it; 2 Department of Astronautical Electrical and Energy Engineering, Sapienza University of Rome, Via Salaria 851-881, 00138 Rome, Italy; susanna.laurenzi@uniroma1.it

**Keywords:** graphene, polymers, sensors, nanocomposites, environmental monitoring, human health monitoring

## Abstract

Graphene-based nanocomposites are largely explored for the development of sensing devices due to the excellent electrical and mechanical properties of graphene. These properties, in addition to its large specific surface area, make graphene attractive for a wide range of chemical functionalization and immobilization of (bio)molecules. Several techniques based on both top-down and bottom-up approaches are available for the fabrication of graphene fillers in pristine and functionalized forms. These fillers can be further modified to enhance their integration with polymeric matrices and substrates and to tailor the sensing efficiency of the overall nanocomposite material. In this review article, we summarize recent trends in the design and fabrication of graphene/polymer nanocomposites (GPNs) with sensing properties that can be successfully applied in environmental and human health monitoring. Functional GPNs with sensing ability towards gas molecules, humidity, and ultraviolet radiation can be generated using graphene nanosheets decorated with metallic or metal oxide nanoparticles. These nanocomposites were shown to be effective in the detection of ammonia, benzene/toluene gases, and water vapor in the environment. In addition, biological analytes with broad implications for human health, such as nucleic bases or viral genes, can also be detected using sensitive, graphene-based polymer nanocomposites. Here, the role of the biomolecules that are immobilized on the graphene nanomaterial as target for sensing is reviewed.

## 1. Introduction

In recent years, significant progress has been achieved in the development of nanocomposite materials with enhanced sensing properties, making them suitable to be used in monitoring devices for a wide range of applications. In particular, the interest in research and fabrication of nanocomposites for sensing has greatly increased due to the possibility to maintain the advantages of the polymer matrix, such as mechanical properties and processability [1], and, at the same time, enhancing its performance by the use of nano-sized fillers [2,3]. Due to the nanometer size of the reinforcing phase, the interface-to-volume ratio is higher than in conventional composites [4]. Nanometric dimensions and extremely high aspect ratios of the tubes and plates used as fillers play a crucial role on the nanocomposite performance, leading to unique properties, such as a low percolation threshold (0.1–2 vol%), a large number of particles per particle volume (10^6^–10^8^ particles/μm^3^), extensive interfacial area per volume of particles (10^3^–10^4^ m^2^/mL), and short distances between particles (10–50 nm at 1–8 vol%) [5]. Indeed, nanocomposites generally show an enhancement in terms of electrical conductivity because of the better compactness of the polymer phase, and therefore show a higher coupling among the nanoparticles through the grain boundaries [6,7].

A crucial aspect concerning polymer-based nanocomposites is the improvement of the nanofiller dispersion in a polymer matrix having different chemical properties. This represents a nontrivial task and is a fundamental requirement to ensure homogeneous properties for this class of composites [8,9,10]. For instance, in the case of graphene nanoplatelets dispersed into a polymer phase, the filler tends to form agglomerates due to the non-covalent van der Waals and π–stacking interactions acting between the nanoparticles, which leads to a loss of performance of the overall nanocomposite. To avoid this, numerous studies have focused on techniques to evaluate and improve the nanofiller dispersion in polymer phases [11,12,13]. A method based on the direct analysis of optical images using a specific MATLAB algorithm was developed to assess the filler dispersion in a quantitative way [11]. A dispersion index was determined by comparing the grayscale optical image with the corresponding uniformly dispersed picture. Gudarzi et al. employed a two-phase reacting system for functionalization of graphene oxide (GO), with the aim of enhancing the dispersion of the GO nanosheets in an epoxy matrix [12]. This procedure revealed a good dispersion and bonding between graphene and epoxy up to 0.5 vol% (~1 wt%) of filler. Goda et al. grafted poly(delta-gluconolactone) from reduced graphene oxide platelets (PGL-g-rGO) in different mass ratios to prepare nanocomposites with a polyvinyl alcohol (PVA) matrix [13]. In this case, the presence of the functional groups on the surface of the PGL-g-rGO filler enhanced the interfacial interaction with PVA, thus improving the filler dispersion.

The aptitude for sensing of graphene/polymer nanocomposites (GPNs) largely derives from the superior conductive properties of graphene. Single-layer graphene shows electrical conductivity up to 6000 S/cm [14,15], which is independent of chirality, and high thermal conductivity up to 5300 W/mK at room temperature [16]. These properties are enhanced by choosing a suitable polymer matrix or substrate, with an optimized interaction at the interphase region with the graphene layer [17,18,19], and by adding specific elements with sensing abilities, such as biomolecules [20,21,22], metals, or metal oxides [23]. Graphene allows detection of gas molecules that attach to or detach from its surface: the adsorbed molecules change the local carrier concentration of graphene, which leads to step-like changes in resistance [24,25]. For this reason, graphene is widely employed for creating sensitive nanomaterials for environmental monitoring applications. In this regard, GPNs allow the detection of harmful gases in the environment, such as ammonia (NH_3_) or nitrogen dioxide (NO_2_) [26,27], and also measurement of the humidity level in specific settings [28,29].

Recently, graphene/polymer nanocomposites have also found applications in radiation monitoring devices, for example those used to assess the levels of ultraviolet (UV) radiation exposure in different environments [30,31,32,33]. GPNs can be employed as UV-sensitive elements for monitoring systems in extreme environments, such as in space [31]. In addition, they can be used in industrial settings characterized by high levels of UV-C radiation, such as in sterilization plants, or to monitor the levels of incoming UV-C radiation in Earth regions that are most at risk of ozone layer depletion.

Graphene-based polymer nanocomposites are also successfully employed in sensing devices for monitoring human health. In this field of application, the high specific surface area and the atomic thickness of the graphene layers play a key role for improving the interaction between carbon atoms and analytes [34]. Moreover, nanocomposite sensors based on graphene sensing elements may guarantee enhanced contact with the skin due to the mechanical flexibility and ultrathin thickness of graphene, eliminating motion artifacts [35]. Furthermore, several biomolecules, such as deoxyribonucleic acid (DNA), enzymes, and antibodies, can be immobilized on the surface of graphene-based sensor platforms that can be used for detecting and monitoring specific analytes [36,37,38].

Other sensing materials can be used to pursue environmental and human health monitoring, such as those based on pure or modified conductive polymers (CPs) [39,40,41], polymer matrix composites (PMCs) filled with carbon nanotubes (CNT) [42,43,44,45], or metal oxides [46,47]. For instance, Xie et al. used pure polyaniline films, polyaniline and acetic acid mixed films, and polyaniline and polystyrene sulfonic acid composites for the detection of nitrogen dioxide (NO_2_) [41]. Other gas sensors were fabricated using polyaniline and metal oxides, such as zinc oxide (ZnO), titanium dioxide (TiO_2_), iron(III) oxide (Fe_2_O_3_), tin(IV) oxide (SnO_2_), tungsten oxide (WO_3_), copper(II) oxide (CuO), and cerium(IV) oxide (CeO_2_) [46]. The incorporation of metal oxides in polyaniline had the effect of improving the poor mechanical strength of the pure polymer and allowed for selectivity towards specific gas molecules [48]. Various biosensors based on polypyrrole were proposed for glucose monitoring [49]. In particular, several studies focused on the fabrication of glucose biosensors using polypyrrole films filled with CNT and glucose oxidase [44,45]. For these nanocomposites, the simultaneous incorporation of the fillers also served to impart biocatalytic and electrocatalytic properties to the sensor.

In this review article, we report on recent trends in the development of graphene/polymer nanocomposites (GPNs) with sensing properties that can be applied for environmental and human health monitoring. The main methods used for the synthesis of graphene are described, focusing on its superior electrical and mechanical properties. The routes for fabricating GPNs are also reported, considering the role of the polymer/filler interface on the final properties of these materials. An overview of GPNs used for detecting harmful gas molecules and/or humidity level in specific environments is presented. Moreover, nanocomposite sensors with the ability to assess UV radiation exposure are presented in view of their application in environmental monitoring devices. Applications of novel GPNs for examining human health parameters are also discussed. The role of biomolecules and how they can be specifically integrated to enhance the sensing properties of the nanomaterials is highlighted.

## 2. The Graphene Nanomaterial: Synthesis and General Properties

**Figure 1:**
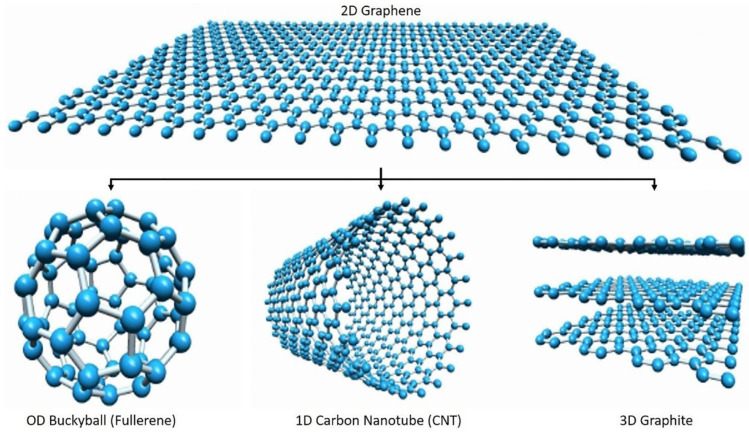
Schematic view of graphene 2D structure (top) and its evolution towards 0D fullerenes by wrap up, 1D nanotubes by rolling, or 3D graphite by stacking. Reproduced with permission from Krishna et al., Constr. Build. Mater.; published by Elsevier, 2021 [50].

Today, graphene represents one of the most attractive nanomaterials employed in the development of functional nanocomposites. It is one of the allotropes of carbon and is composed of one-atom-thick planar sheets of sp^2^-bonded carbon atoms that are densely packed in a honeycomb crystal lattice. This 2D structure can be wrapped up into 0D fullerenes, rolled into 1D nanotubes, or stacked into 3D graphite, as shown in Figure 1 [50]. The exceptional properties shown by graphene justify its increasing use as a functional filler, especially for polymer-matrix-based nanocomposites [51,52]. In particular, it possesses peculiar electrical characteristics, such as an anomalous quantum hall effect and a high electron mobility at room temperature (23 × 10^4^ cm^2^/Vs) [53,54] and shows excellent mechanical performance [55]. Specifically, its high tensile strength, elasticity, Young’s modulus (~1 TPa) and spring constant are mainly due to its hexagonal lattice structure, with the sp^2^ bonds conferring stability and opposing in-plane deformation [56]. In addition, graphene shows high thermal conductivity (~5000 W/mK) [16], and its field of application is further extended by the possibility to functionalize it chemically. Several biological applications involving graphene highlighted its biocompatibility [57,58,59] (although this aspect is currently the subject of extensive investigations due to the possible toxicity of nanomaterials).

Different approaches are currently used for the synthesis of graphene. These can be grouped into two main categories that are referred to as bottom-up and top-down methods [60,61]. Typically, the bottom-up approaches allow small-scale production of graphene, characterized by high quality and large size sheets, starting from carbon compounds. These methods include techniques such as carbon vapor deposition (CVD), arc discharge, epitaxial growth on silicon carbide (SiC), self-assembly, and reduction of CO_2_ [61], and allow production of both monolayer and multiple-layer graphene, as reported in Table 1. Conversely, the top-down methods allow large-scale production of graphene, pristine or functionalized, in the form of small particles. Therefore, this approach is particularly suitable to synthetize graphene to be used as filler in polymer nanocomposites. It involves the separation of graphene directly from graphite or graphite derivatives and includes several methods, which are summarized in Table 2, such as exfoliation or super acid dissolution of graphite, solvothermal reduction or chemical reduction of graphite oxide, and thermal exfoliation/reduction of graphite oxide.

**Figure 2:**
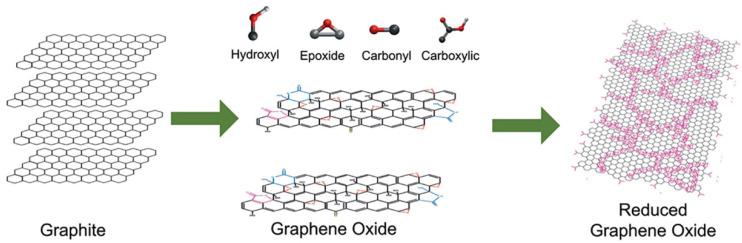
Schematic representation of the structure of graphene oxide and reduced graphene oxide showing functional groups at the edges. Reproduced with permission from Bonaccorso et al., Mater. Today; published by Elsevier, 2012 [83].

In general, the commonly referred to “graphite oxide” (GO) is made of graphene oxide sheets stacked with an interlayer spacing of between 6 and 10 Å, depending on the water content [79]. According to the Lerf–Klinowski model [79,80,81,82], graphene oxide can be described as pristine aromatic “islands” separated from each other by aliphatic regions containing epoxide and hydroxyl groups and carbon–carbon double bonds. Typically, hydroxyl and epoxy groups can be detected at higher concentrations on the basal plane of graphene oxide, whereas carbonyl and carboxylate acid groups are found at the sheet edges [80]. A representation of graphene, graphene oxide, and reduced graphene oxide structures is showed in Figure 2 [83].

The thermal exfoliation of GO allows separation of the graphene oxide sheets and reduction of the oxygen content, generally leading to restoration of the ability to conduct electricity [84]. In particular, GO can be reduced and exfoliated simultaneously upon rapid heating, which induces the thermal decomposition of the oxygen-containing functional groups due to the pressure of the gas products, in particular CO_2_, that builds up instantaneously between the sheets [76,84,85]. The obtained graphene can be dispersed in polar organic solvents due to the polar oxygen-containing functional groups remaining on it and due to its wrinkled nature preventing the sheets from restacking [76,84,86]. The oxygen-to-carbon element ratio and the electrical conductivity of the resulting graphene can be modulated depending on the time and temperature adopted during the process [87]. For instance, high quality graphene with fewer structural and topological defects was obtained at lower process temperatures, under vacuum, in the presence of an accelerating agent (such as H_2_ or HCl), or with microwave or irradiation assistance [88,89].

The chemical functionalization of graphene represents a valid approach to improving the interaction between the filler and the polymer matrix, further increasing the solubility of the reinforcement [90,91,92,93]. The process involves functional groups that can be small molecules [94] or polymer chains [95], and both covalent and non-covalent functionalization can be carried out [17]. In particular, covalent functionalization can be performed at the end of the graphene sheets or on their surface and involves rehybridization of one or more sp^2^ carbon atoms into the sp^3^ configuration by nucleophilic substitution, electrophilic addition, condensation, or addition [96]. Concerning the non-covalent functionalization, it allows the connection between the molecules without involving chemical bonds and generally requires the physical adsorption of suitable molecules on the graphene surface [97]. Specifically, these molecules wrap around graphene by means of van der Waals forces and can involve π–π interactions, electrostatic attraction, adsorption of surfactants, and polymer wrapping [98,99,100,101].

## 3. Graphene/Polymer Nanocomposites: Fabrication and Properties

Graphene-based nanocomposites with a polymer matrix are commonly fabricated following three different methods: solution blending, in situ polymerization, and melt mixing [102,103,104]. The most common technique is solution blending [102], which involves solubilization of the polymer in a suitable solvent and mixing with graphene to form a homogeneous dispersion. Generally, polymers such as polystyrene, polycarbonate, polyacrylamide, polyimides, and poly(methyl methacrylate) are mixed with graphene oxide [105,106,107,108], which can be previously functionalized with isocyanates, alkylamine, or alkyl-chlorosilanes in order to improve its dispersibility in organic solvents.

The fabrication of GPNs by in situ polymerization is based on the polymerization of the matrix in the presence of the selected filler, starting from a mixture of monomer and reinforcement [102,103,104]. Typically, this approach allows the obtainment of a good grade of dispersion of graphene-based nanofillers, avoiding the need of preliminary exfoliation [104]. In the melt mixing technique, the filler is dispersed in the polymer matrix, exploiting high temperatures and shear forces [74]. The polymer phase is melted at a high temperature, thus facilitating the dispersion or intercalation of the graphene oxide nanoplatelets without the use of organic (often toxic) solvents.

The main polymers employed in the manufacture of graphene-based nanocomposites are reported in Table 3, with the indication of the method used for the fabrication and the type of property enhanced by the presence of the filler.

The properties of the GPN nanocomposites are strictly related to the spatial distribution and alignment of the graphene nanofiller and to its interfacial adhesion with the polymer phase. In particular, GPNs with low loadings of functionalized graphene sheets generally exhibit a shift in the glass transition temperature [107] compared to that of uncharged polymer. This behavior can be ascribed to a reduced mobility of the polymer chains at the interface between the filler and the matrix [122,123]. Therefore, the constraint applied on the chains can directly induce an increase in glass transition temperature [124,125].

In terms of thermal conductivity, the performance of GPNs can be evaluated by referring to the 2D geometry of the graphene fillers. These are characterized by lower interfacial thermal resistance that provides higher thermal conductivity to the host polymer matrix [51,126]. Nevertheless, the 2D structure can be a source of anisotropy in the nanocomposite arrangement, for which the in-plane thermal conductivity can be as much as ten times higher than the cross-plane conductivity [127]. This is typically evaluated following the percolation theory, therefore considering phonons as the main mode for thermal conduction in polymers. Covalent bonding between the filler and the polymer matrix can reduce phonon scattering at the interface, leading to an overall enhancement of the GPN thermal conductivity [128].

The electrical conductivity behavior of GPNs can be analyzed by considering the influence of different factors and their overall effect. In particular, the characteristics of the specific graphene-based filler, such as its aspect ratio and morphology, as well as its inter-sheet junction, can affect the electrical performances of GPNs [51,129]. In the same way, processing, dispersion, and the related state of aggregation and alignment of the nanoparticles coalesce to determine the electrical behavior of the resulting nanocomposite [51,130]. Several theoretical models and experiments have aimed to assess the role of nanofiller shape, geometry, and state of dispersion on the percolation threshold of graphene/polymer nanocomposites [131,132].

**Figure 3:**
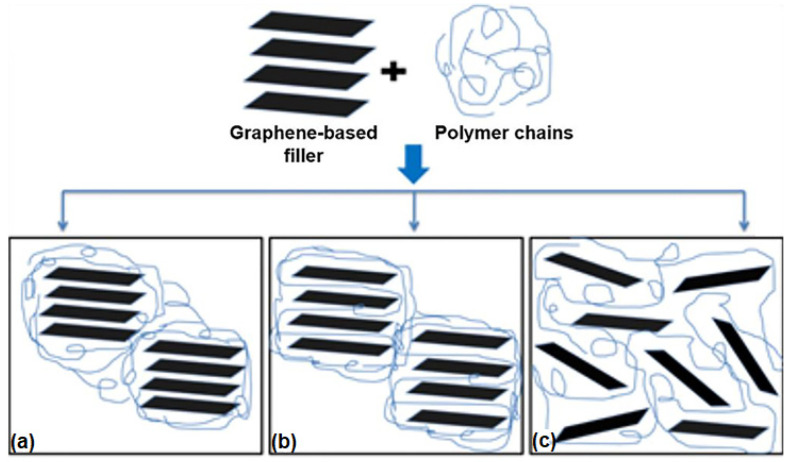
Different arrangements of the filler in graphene-based nanocomposites: (**a**) separated, (**b**) intercalated, and (**c**) exfoliated state.

As mentioned above, the overall performance of polymer-based nanocomposite materials can be related to the quality and stability of the polymer/nanofiller interphase region. Typically, the physical and mechanical properties and the chemical composition of this region are different from those of the bulk polymer matrix [133,134]. In the case of an interphase stiffer than the surrounding polymer, this can result in higher overall stiffness and strength of the composite, but with lower resistance to fracture [135]. The interphase properties can also affect the mechanical behavior of the nanocomposites depending on the morphology and size of this region. In fact, several studies show that its thickness can be tailored to achieve both higher strength and improved toughness of the resulting nanocomposite [18,136,137]. Force-modulation atomic force microscopy (AFM) and nanoindentation are techniques that are commonly used to investigate the interphase and its properties [133,138]. In particular, AFM phase imaging is currently considered a useful tool to evaluate the thickness and the relative stiffness of the interphase, since it involves much lower interaction forces between the probe and the sample than force modulation or nanoindentation [135]. The arrangement of graphene-based fillers inside the polymer matrix is also investigated in order to assess the state of dispersion at the microstructural level and its impact on the nanocomposite properties. Results reveal that graphene-based fillers, such as graphene oxide or graphene nanoplatelets, can arrange differently in the host polymer, originating structural states that can be classified as stacked, intercalated, or exfoliated [104], as showed in Figure 3.

The intercalated state can be considered as a particular type of stacked structure, which is characterized by greater interlayer spacing (but still within a few nanometers) [139]. Generally, in the exfoliated structure, graphene nanoplatelets have the largest interfacial contact with the polymer matrix, and this allows improvement in the performance of the composite in different ways [102,104]. Due to the interactions with the matrix, the exfoliated phase can exhibit a curved shape. In this case, the rumpled shape assumed by the filler can result in mechanical interlocking, acting as a possible mechanism of strengthening. The compatibility between the host polymer and the nanoplatelets is one of the major factors determining the filler morphology in the matrix: the nanoplatelets are characterized by a more extended conformation for high polymer/filler affinity or, conversely, a crumpled conformation when the affinity decreases [140]. Finally, the processing method used to fabricate the nanocomposite also affects its microstructure to a great extent: solution mixing or in situ polymerization generally induce an exfoliated and randomly oriented status to the nanoplatelets, whereas the melt mixing technique generates a more oriented and intercalated or stacked structure of the nanoplatelets [141].

## 4. Applications of Graphene/Polymer Nanocomposites in Sensing Devices

The enhanced properties of graphene/polymer nanocomposites and the possibility to tailor their performance toward more specific applications make them suitable materials for sensing. In this perspective, the ability to detect an external stimulus, whether of a chemical, physical, or biological nature, and give information about it can be developed through accurate selection and successive engineering of the starting materials. Graphene is one of the most promising materials for sensing applications, and its properties are widely employed singularly or functionally combined with other suitable materials [142,143,144]. Metal oxide nanoparticles, for instance, are usually coupled with graphene, graphene oxide, or reduced graphene oxide for large-scale production of sensing devices in different fields, from environmental pollution to clinical and pharmacological detection [145]. Another effective integration derives from the coupling of graphene and biological molecules [146,147], thus realizing hybrid nanomaterials with enhanced functional properties that can be wisely employed for sensing applications.

### 4.1. Applications of GPNs in Environmental Monitoring: Gas and Humidity Sensors

Graphene oxide and reduced graphene oxide are successfully used to fabricate gas-sensitive graphene/polymer nanocomposites due to their ability to entrap gas molecules that in turn cause a variation to the conducting properties [26,148,149,150]. In fact, oxidizing and reducing gases interact with graphene in different ways, leading to carrier generation or carrier annihilation with a change in the sensor resistance or current [151,152]. The addition of metal (nano)particles to graphene oxide can enhance its response to gas molecules [152]. The choice of a specific metal can be performed by evaluating the best solid–gas interactions depending on the nature of the analyte. For instance, palladium exhibits a significant affinity for hydrogen, promoting the dissociation of its molecules into atoms [153], whereas nickel is more suitable for carbon monoxide detection as it forms nickel carbonyl with a sufficiently low activation energy [154]. Therefore, graphene can be decorated with metallic nanoparticles and opportunely coupled to polymers to fabricate GPN nanocomposites that are able to detect specific gas molecules.

**Figure 4:**
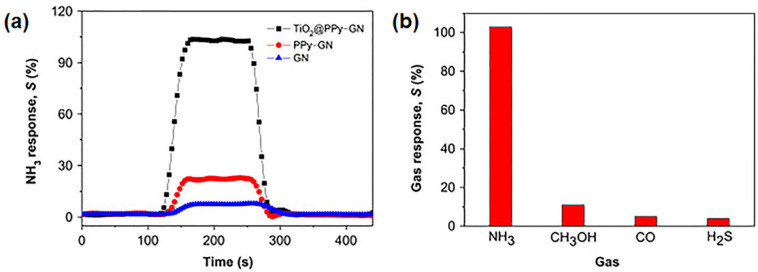
(**a**) Gas response of GN, PPy/GN, and TiO_2_/PPy/GN samples to NH_3_ (50 ppm) at room temperature; (**b**) Response of TiO_2_/PPy/GN nanocomposite to different gases at a fixed concentration of 50 ppm. Reproduced with permission from Xiang et al., Ceram. Int.; published by Elsevier, 2015 [26].

A sensor based on polypyrrole (PPy) and graphene nanoplatelets (GN) decorated with titanium dioxide (TiO_2_) nanoparticles was developed for detecting ammonia (NH_3_) gas molecules [26]. The nanocomposite was synthesized by a sol–gel process combined with in situ chemical polymerization. The NH_3_-sensing properties were investigated for GN, PPy/GN thin films, and TiO_2_/PPy/GN nanocomposites under the same conditions, demonstrating the highest sensitivity and the fastest response for the TiO_2_/PPy/GN samples (Figure 4a). Results show a good electrical-resistance response for the TiO_2_/PPy/GN nanocomposite, which is able to detect NH_3_ molecules at room temperature with reproducibility and excellent selectivity (Figure 4b).

**Figure 5:**
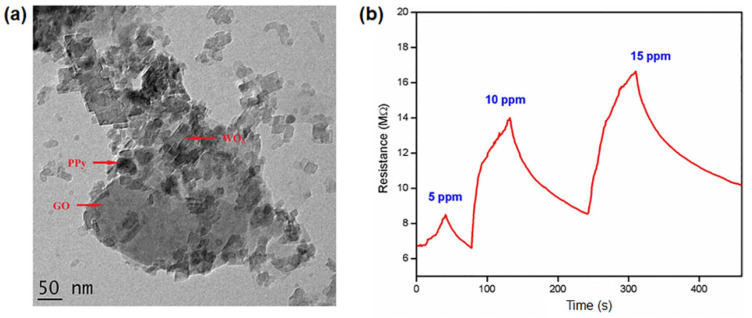
(**a**) Morphology of PPy–GO–WO_3_ nanocomposite investigated by transmission electron microscopy; (**b**) electrical response of the PPy–GO–WO_3_ nanocomposite to NH_3_ gas (5–15 ppm). Reproduced with permission from Albaris et al., Mater. Sci. Eng. B: Solid-State Mater. Adv. Technol.; published by Elsevier, 2020 [148].

Another type of graphene/polymer nanocomposite sensitive to NH_3_ was realized using polypyrrole (PPy) and tungsten oxide (WO_3_). A chemical oxidative polymerization of polypyrrole in the presence of GO–WO_3_ filler was used to realize the nanocomposite [148]. The uniform distribution of the WO_3_ nanoparticles on the PPy-decorated GO nanosheets was confirmed by transmission electron microscopy (Figure 5a). The nanocomposite showed an improved sensitivity (by 58%) with reduced response time (50 s) and recovery time (120 s) in an environment at room temperature (30 °C) and 50% relative humidity (RH). The lower detection limit was measured at 5 ppm (Figure 5b).

A gas-sensitive polymer, such as polyaniline (PANI), and reduced graphene oxide (rGO) nanosheets were used for preparing nanocomposite films for ammonia (NH_3_) detection [155]. The responsivity of the PANI-rGO nanocomposite as an NH_3_ gas sensor revealed consistent values at different relative humidity conditions and could be used under highly humid environments. Generally, graphene/PANI nanocomposites are synthetized by in situ chemical polymerization and solution mixing. In particular, in situ chemical polymerization allows more uniform dispersion of graphene oxide (GO), creating strong interactions between the filler and the polyaniline matrix [150].

Polyaniline–graphene nanoplatelet (PANI–GN) nanocomposites were fabricated for detecting toluene and benzene gases at ambient temperature [149]. They were synthesized with different amounts of graphene in the polymer matrix using in situ polymerization of aniline monomers in the presence of GNs. The sensitivity was dependent on the content of graphene in the PANI matrix: nanocomposites with 6 wt% of graphene nanoplatelets showed a maximum sensitivity of 90% and 80% for toluene and benzene gases, respectively. In particular, the PANI–GN nanocomposites showed higher sensitivity and decreased response and recovery times towards toluene as compared to benzene, likely due to the higher diffusion rates of toluene gas molecules into the nanocomposite. PANI–GN nanocomposites could be used as conductometric sensors for the detection of toluene at lower concentrations than benzene.

**Figure 6:**
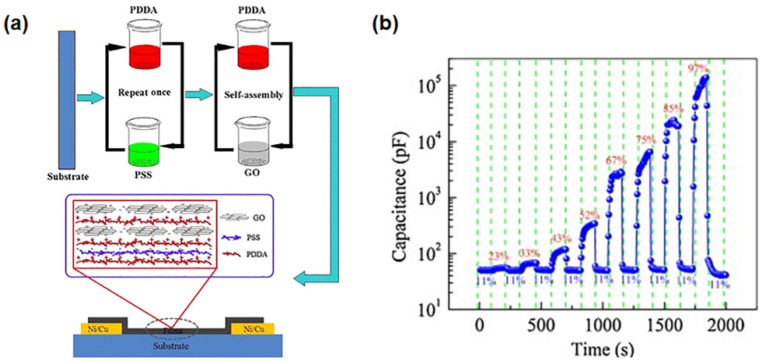
(**a**) Schematic representation showing the layer-by-layer fabrication of GO/PDDA nanocomposite films. (**b**) Variation of the capacitance value for the GO/PDDA sensors upon switching the relative humidity levels in the range of 11–97%. The exposure/recovery cycles were performed with an exposure interval of 125 s followed by a recovery interval of 125 s at 11% RH. Reproduced with permission from Zhang et al., Sens. Actuators B Chem.; published by Elsevier, 2014 [29].

GO/poly(diallyldimethylammonium chloride) (PDDA) nanocomposite films were deposited on a flexible polyimide (PI) substrate and used as a humidity sensor [29]. The multilayer GO/PDDA nanocomposites were realized using the layer-by-layer self-assembly technique as illustrated in Figure 6a. The sensing properties were investigated at relative humidity levels in the range of 11–97%. Several exposure/recovery cycles were performed using an exposure interval of 125 s followed by a recovery interval of 125 s at 11% RH. Figure 6b shows the variation of capacitance for the GO/PDDA films upon switching the humidity level. The area between two adjacent dotted lines identifies each cycle, and an increase in the capacitance value is observed with the rising of RH in the range of 11–97%. The sensing properties were investigated at different relative humidity levels in the range of 11–97%. These sensors exhibited an extremely high response, reaching unprecedented values of 265,640%. The sensing mechanism of the GO/PDDA nanocomposite was analyzed using complex impedance spectra and bode diagrams, which gave an indication of the water molecule permeation into the mesopores of the multilayer film. Its response and recovery times make this nanocomposite material suitable for humidity detection in various contexts, including measuring human breath.

**Figure 7:**
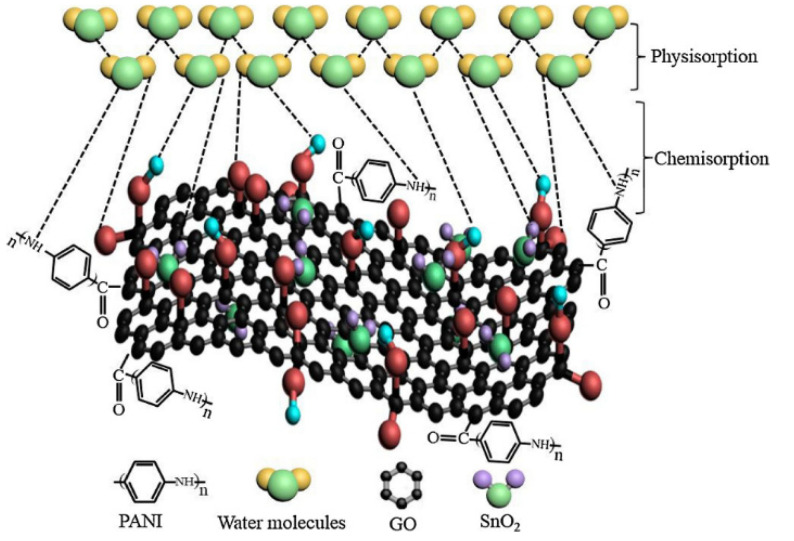
Mechanism of humidity sensing of the GO/SnO_2_/PANI nanocomposite involving physisorption and chemisorption of the water molecules. Reproduced with permission from Zhang et al., Sens. Actuators B Chem.; published by Elsevier, 2018 [28].

A quartz-crystal microbalance (QCM) resonator comprised of a GO/tin dioxide/polyaniline (GO/SnO_2_/PANI) nanocomposite was synthetized via in situ oxidative polymerization and used as a humidity sensor [28]. The humidity-induced frequency changes of the QCM sensor were investigated upon exposure to different relative humidity (RH) levels at room temperature. High sensitivity of 29.1 Hz/%RH was observed over a wide range of 0–97% RH, with short response and recovery times of 7 s and 2 s, respectively. The humidity sensing mechanism, which is schematically depicted in Figure 7, was analyzed using the Langmuir adsorption isotherm model. Water molecules adsorb on the GO/SnO_2_/PANI nanocomposite, linking to the hydroxyl, carboxyl, and epoxy groups that are attached on GO, the amino groups on PANI, and the surface vacancies on the SnO_2_ nanoparticles. In particular, water molecules are firstly chemisorbed on the coated film at low RH, and then they are physisorbed by double hydrogen bonding. At high RH, second-layer water molecules are physisorbed through single hydrogen bonding.

**Figure 8:**
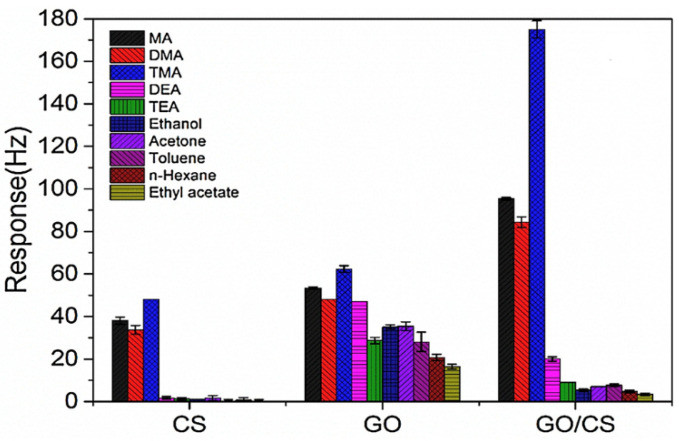
Comparison of the responses of pure CS-, GO-, and GO/CS-coated QCM sensors to 50 ppm of various organic vapors. Reproduced with permission from Zhang et al., Sens. Actuators B Chem.; published by Elsevier, 2017 [156].

Nanocomposites based on graphene oxide and chitosan (GO/CS) were used as sensing material of quartz crystal microbalance (QCM) sensors to detect amine vapors [156]. The GO/CS nanocomposite was developed in a porous mesh structure made of interconnected nanofibers with diameters of 50 nm. The response of the GO/CS coated sensor was compared to that of the sensors functionalized with pure CS and pure GO after exposure to different organic vapors (Figure 8). At room temperature, the GO/CS nanocomposite showed high sensitivity to aliphatic amines such as methylamine (MA), dimethylamine (DMA), and trimethylamine (TMA), with sensitivity values of 2.7, 2.3, and 4.8 Hz/ppm, respectively. The sensing mechanism is based on the adsorption/desorption of the amine vapors due to the hydrogen-bonding interaction of the protonated amine with the hydroxyl sites of the GO/CS film. The sorption properties and the sensing mechanism were described using the linear solvation energy relationship (LSER) model [157]. The LSER model defines the sorption properties in the form of sorbent and solute pairs, determining a connection between the sorption properties of the sensing material and the chemical and physical parameters of the analytes. The experimental results revealed reversibility, repeatability, and long-term stability for the GO/CS nanocomposite.

Another interesting class of graphene/polymer nanocomposites for gas-sensing applications is based on conductive polymers, such as poly(3,4-ethylenedioxythiophene) (PEDOT). Chemically modified graphene/poly(3,4-ethylenedioxythio-phene):poly(styrenesulfonate) (PEDOT:PSS) nanocomposite films were fabricated for hydrogen (H_2_) sensing [158]. The sensing behavior of rGO/PEDOT:PSS nanocomposites can be considered the same of a n-type material, whereas GO/PEDOT:PSS shows p-type characteristics. GO/PEDOT:PSS exhibited better H_2_ gas-sensing properties than rGO/PEDOT:PSS. This result may be related to the poor distribution of the nanofillers in the rGO/PEDOT:PSS samples. The sensitivity, response time, and recovery time of the GO/PEDOT:PSS sensors toward H_2_ molecules (100 ppm) were 4.2%, 30 s, and 25 s, respectively. In the work by Yang et al., porous poly(3,4-ethylenedioxythiophene) (PEDOT) nanostructures were deposited on reduced graphene oxide (rGO) films, resulting in sensing platforms for nitrogen dioxide (NO_2_) detection [27]. The sensing performance of these nanocomposites was compared to that of gas sensors based on bare rGO and on common rGO/PEDOT composites. Results revealed that the rGO/porous PEDOT samples possess an enhanced gas adsorption and desorption property, which can be ascribed to the high surface area and the porous nanostructure of these nanocomposites.

### 4.2. Applications of GPNs in Environmental Monitoring: UV Radiation Sensors

On Earth, exposure to UV radiation, particularly to that in the dangerous UV-C region, is limited by several factors, most notably the presence of the ozone layer that blocks the shorter wavelengths (100–280 nm) that are typical of the UV-C band. On the other hand, the space environment is characterized by intense and unfiltered UV radiation, which accounts for about 10% of the total electromagnetic radiation originating from the Sun [159].

Miniaturized UV sensors can be employed to monitor hazardous exposures during working activities involving artificial sources of radiation without interfering with normal activities [160,161]. Some industrial applications, such as sterilization processes, rely on the use of highly damaging UV-C radiation, necessitating wearable devices that can reliably detect and quantify any accidental exposure to workers [162]. In addition, an enhanced UV-sensing system would be useful to detect small amounts of incoming UV-C radiation and to correlate these measurements with ozone layer depletion, particularly in regions that are most at risk, such as those near the equator.

The nanomaterials involved in the engineering of UV sensors typically include structures such as zinc oxide and silver nanoparticles [163,164,165]. In this field, graphene properties can be exploited to improve carrier transport and UV absorption and therefore the photo-response of the sensors. Nanocomposites based on zinc oxide nanowires and reduced graphene oxide [166,167] show enhanced performance compared to photodetectors based on nanowires made of pure zinc oxide. In particular, graphene nanosheets allow the development of conductive networks and enhanced photo-response due to the large interface regions between graphene and zinc, which prevents carrier recombination and facilitates its transport [166]. Reduced graphene oxide was also combined with tungsten oxide nanodiscs to fabricate UV-sensitive composite materials [168]. Tungsten oxide shows an indirect, large, energy-band gap that makes it a good candidate for UV detection. Nevertheless, only a few studies [153,169] have focused on its employment for this purpose, probably due to its very slow response time. The coupling of tungsten oxide with reduced graphene oxide led to a response time on the order of milliseconds [168], with the improvement attributed to the carrier transport efficiency of graphene.

**Figure 9:**
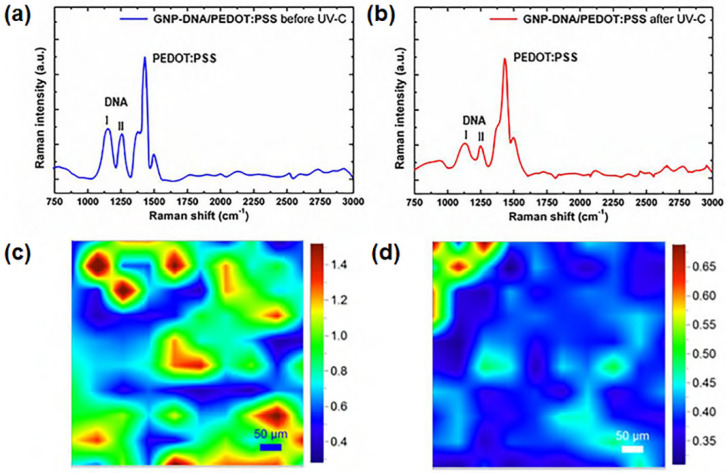
Raman analysis of GNP-DNA/PEDOT:PSS film (**a**,**c**) non-irradiated and (**b**,**d**) after UV-C exposure for 6 days. (**a**,**b**) Raman spectra; (**c**,**d**) Raman maps obtained using the intensity of the DNA I peak normalized to the intensity of the main PEDOT:PSS peak as contrast parameter. Reproduced with permission from Toto et al., Appl. Surf. Sci.; published by Elsevier, 2020 [33].

Several studies have demonstrated that graphene nanoplatelets (GNs) can be successfully functionalized with DNA molecules in order to obtain UV-sensitive complexes that can be further embedded in a polymer matrix [30,31,32,33,170,171,172]. In this way, novel graphene/polymer nanocomposites with UV-sensitive properties are obtained. For example, UV-sensitive nanocomposite films were prepared by integrating DNA-modified graphene nanoplatelets with a polymer matrix made of poly(3,4-ethylenedioxythio-phene):poly(styrenesulfonate) (PEDOT:PSS) [32,33]. The superior electrical properties and the mechanical strength of graphene were used to enhance the properties and stability of the PEDOT:PSS, whereas the DNA molecules were sensitive to UV and were shown to have an exfoliating effect on the GNs in aqueous solution. The high specific surface area of the nanoplatelets favors the formation of a large number of non-covalent interactions between the amines of the DNA molecules and the carboxyl groups of the nanoplatelets [173,174]. Films were exposed to UV-C radiation and different techniques were used to evaluate their response under UV exposure. Results were useful to assess the ability of these nanocomposites to detect an absorbed dose. Raman microscopy mapping was used to investigate the chemical modifications caused by the radiation and the role of each component of the films in the overall response of the nanocomposites (Figure 9) [33]. These graphene–DNA/PEDOT:PSS films could be used in ultra-small and lightweight UV sensor devices for monitoring in space or for industrial settings on Earth that are characterized by high levels of UV-C radiation. Moreover, electrical resistance tomography (ERT) was used to investigate the conductivity changes occurring at the surface of the nanocomposite coatings during UV irradiation, providing in situ monitoring of UV-induced degradation in composite materials and structures [30].

Graphene–DNA complexes were also embedded in polydimethylsiloxane (PDMS) matrices, fabricating free-standing thin films and 3D materials with improved conformability and reduced size [31,170,172,175]. PDMS is well known for its chemical inertia, biocompatibility and flexibility, which make it an excellent candidate for wearable sensors. Furthermore, PDMS exhibits high optical transparency to UV radiation with good transmittance in the UV-C band (above 240 nm) [176], allowing major exposure of the incorporated graphene–DNA filler during irradiation. The properties of graphene–DNA/PDMS nanocomposite films were investigated before and after exposure to UV-C radiation, and the effect of different amounts of filler was evaluated. In addition, these nanocomposites were also tested as free-standing 3D materials in a simulated space environment [31], and they showed good stability in terms of thermal and wettability properties with a considerable electrical response to irradiation.

### 4.3. Applications of GPNs in Human Health Monitoring

Many sensors based on nanocomposite materials find applications in the monitoring of human health parameters [177,178]. In this regard, the sensing devices need to be comfortable to wear, biocompatible, and lightweight [179,180,181]. They need to interface with the human body, concurrently showing high selectivity and sensitivity in detecting and quantifying specific signals or analytes.

Graphene, graphene oxide, and chemically modified graphene are widely employed to fabricate nanocomposites suitable for detecting biological analytes, such as uric acid and ascorbic acid [182], hydroquinone and catechol [183], and nucleic bases [184,185]. The presence of functional groups on the nanocomposite surface is fundamental to creating hydrogen bonds with the analytes, so the strength of these bonds and the distance between the interaction sites and the reaction center make possible the discrimination of the analytes.

DNA molecules can be immobilized on a graphene surface by physical adsorption or by chemical binding, thus creating sensitive platforms where each binding event with the analyte can be detected through the changes of the electric or electrochemical properties of these platforms [186,187]. Noncovalent interactions can be promoted through physical adsorption, involving π–π stacking interactions between the DNA nucleobases and the aromatic surface of graphene. In particular, in the case of single-stranded DNA (ssDNA), stable aqueous dispersions of graphene/DNA can be obtained without traces of sedimentation for months [173]. Double-stranded DNA (dsDNA) is also used as a dispersing agent for graphene nanoplatelets. However, in this case less stable aqueous solutions are obtained due to the weaker hydrophobic interactions arising from the base pairing of the nucleobases. Nevertheless, graphene/dsDNA affinity can be significantly enhanced by further functionalizing graphene oxide with polar groups, which are able to establish electrostatic interactions with the DNA bases. The immobilization of DNA on graphene through covalent bonds is generally carried out after functionalizing the DNA with amino groups, which are able to interact with the graphene oxide surface via carbodiimide chemistry [188]. In particular, amine-terminated ssDNA can be linked to the surface of graphene oxide directly or through the involvement of specific molecules that act as carriers.

**Figure 10:**
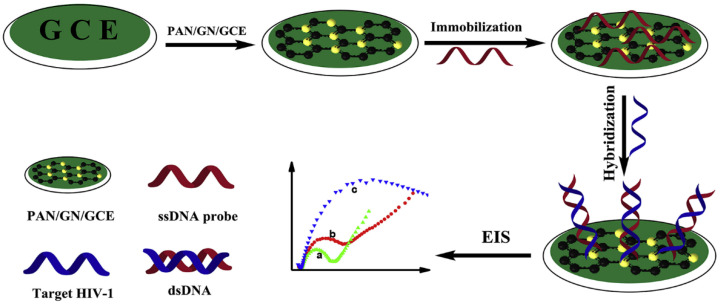
Schematic illustration of the fabrication of HIV-1 gene sensor based on PAN/GN/DNA nanocomposite. The inner graph shows cyclic voltammograms of (a) PAN/GN/GCE, (b) ssHIV/PAN/GN/GCE, and (c) ssHIV/PAN/GN/GCE hybridized with 1 × 10^−11^ M of target HIV. Reproduced with permission from Gong et al., J. Mater.; published by Elsevier, 2019 [20].

Single-stranded DNA was covalently immobilized on a polyaniline/graphene (PAN/GN) nanocomposite, which was applied onto a glassy carbon electrode (GCE) and used for HIV-1 gene detection [20]. The procedure is described schematically in Figure 10. In particular, the negatively charged phosphate backbone of HIV-1 binds to the sensitive surface via π–π* stacking interactions. The hybridization between the ssDNA probe and the HIV-1 target generates double-stranded DNA (dsDNA), which increases electron-transfer resistance in proportion to the concentration of the gene. The sensitivity and the selectivity of this nanocomposite were tested, and a low detection limit of 1.0 × 10^−16^ M for the HIV-1 target was measured.

The development of new composite materials using biomolecules, such as enzymes, has allowed further extension into the fields of sensing applications in medical diagnosis and bio-industrial analysis. Several sensitive nanocomposites have been realized using natural polymers, such as gelatin, alginate, and chitosan, as matrix due to their intrinsic biodegradability and biocompatibility that make them suitable for biomedical applications [189]. In particular, chitosan has been combined with graphene to develop nanocomposite materials with sensing properties useful for monitoring human health [190,191,192,193].

**Figure 11:**
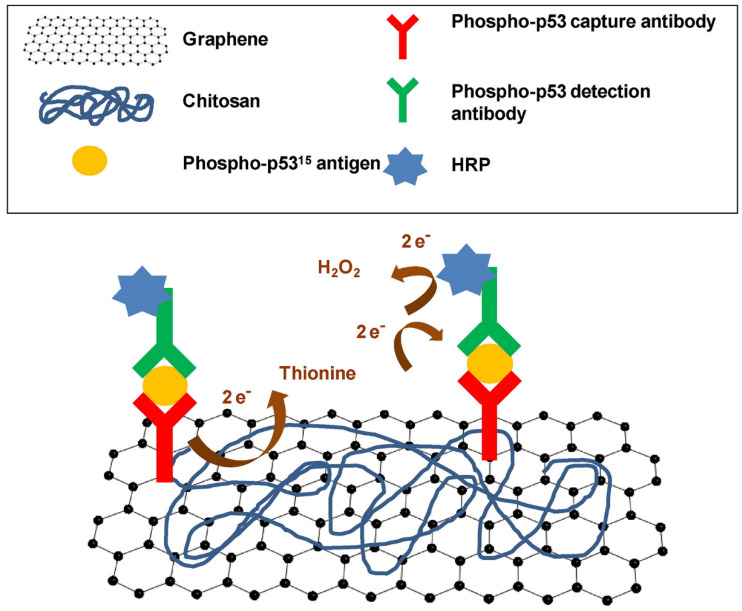
Schematic representation of sensitive nanocomposite based on graphene and chitosan-modified screen-printed carbon electrode (SPCE). Reproduced with permission from Orecchioni et al., Adv. Drug Deliv. Rev.; published by Elsevier, 2016 [193].

Xie et al. developed an immunosensor based on graphene and chitosan-modified screen-printed carbon electrode (SPCE) (Figure 11) [21]. The phospho-p53 capture antibody was adsorbed on the surface of the graphene–chitosan/SPCE. A sandwich immunocomplex was formed between the targeted phospho-p53^15^ antigen, the phospho-p53 capture antibody, the antigen, and the biotinylated phospho-p53^15^ detection antibody, which was previously marked with horseradish peroxidase (HRP). The high surface area of graphene allowed the immobilization of a large amount of capture antibody, increasing the sensitivity of this nanocomposite immunosensor.

Nanocomposite films based on glucose oxidase (GOD), platinum (Pt), functional graphene sheets (FGS), and chitosan were developed for glucose sensing [191]. The electrocatalytic action of FGS and Pt nanoparticles towards hydrogen peroxide (H_2_O_2_) was exploited to obtain a sensitive biosensor with a detection limit of 0.6 μM for glucose. The performance of this type of sensor can be ascribed to the large surface area and the fast electron transfer of graphene and Pt nanoparticles. This sensor showed good reproducibility and long-term stability, with negligible response to other compounds such as ascorbic acid and uric acid. The GOD/Pt/FGS/chitosan-sensitive nanocomposite can be useful for both clinical and home-care devices for rapid monitoring of glucose.

Glucose sensing was also performed with composite films made of graphene, chitosan and uric acid, which were deposited onto glassy carbon electrodes [192]. A molecularly imprinted electrochemical sensor was obtained, and its sensitivity mechanism was analyzed by electrochemical impedance spectroscopy and chronocoulometric methods. A comparison between graphene-doped and undoped sensors was carried out, with results demonstrating an improvement in terms of sensitivity due to the high surface area and good electronic conduction of graphene.

Sensitive films based on EDTA-modified reduced graphene (EDTA-RG) and Nafion were fabricated and tested as dopamine detectors [194]. Graphene was chemically modified by silanization using *N*-(trimethoxysilylpropyl) ethylenediamine triacetic acid (EDTA-silane). The selectivity was investigated by using dopamine and ascorbic acid. Experimental tests demonstrated that the sulfuric groups of Nafion and the carboxylic groups of EDTA-RG interfere with the diffusion of ascorbic acid, thus enabling the selective detection of dopamine.

**Figure 12:**
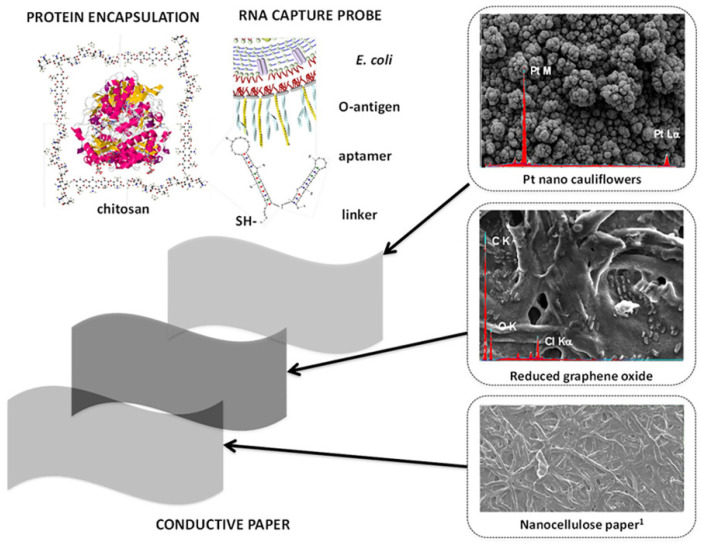
Conductive paper made of graphene–nanocellulose strips decorated with platinum nano-cauliflowers. The paper can be functionalized with glucose oxidase encapsulated in chitosan or RNA for biosensing purposes. Reproduced with permission from Burrs et al., Biosens. Bioelectron.; published by Elsevier, 2016 [195].

More recently, biosensing has seen advances towards more complex structures that are able to enhance the overall sensitivity of the detecting surface. A sensing composite material was realized using fractal nanoplatinum with a cauliflower-like morphology, which was developed on a reduced graphene oxide paper [195]. Platinum was electrodeposited on the graphene–nanocellulose sheets using pulsed sonoelectrodeposition. As a result, a conductive nanocomposite paper with a highly electroactive surface was obtained and then functionalized using glucose oxidase (via chitosan encapsulation) or RNA aptamer (via covalent linking) as depicted in Figure 12. In this way, the material’s sensitivity towards glucose or Escherichia coli bacteria can be activated. Depending on the enzyme selected, good performance in terms of sensitivity and response times were obtained.

## 5. Summary and Final Remarks

Recent trends in the development of graphene-based polymer nanocomposites (GPNs) with sensing properties were examined, with emphasis on applications in the fields of environmental and human health monitoring. In this review article, the methods that are traditionally used to synthetize and functionalize the graphene filler were included. These methods can be classified into two main categories, namely bottom-up and top-down approaches, and allow for the production of graphene sheets with various dimensions (thickness and lateral size) and purity. Next, we discussed methods for the fabrication of GPNs, taking into account the role that the interactions between the polymer and the graphene nanomaterial have on the composite’s final properties. Typically, in situ polymerization is a good choice for obtaining homogeneous nanocomposites by creating a polymer network directly around the fillers, thus avoiding energetic methods—which might cause damage to the conductive capability of graphene—for dispersion. For environmental monitoring, graphene/polymer nanocomposites with metallic or metal oxide nanoparticles immobilized on the graphene filler possess the ability to detect harmful gas molecules (ammonia, benzene, and/or toluene) or measure humidity. Graphene-based nanocomposites with UV-sensing properties have recently been developed by immobilizing DNA strands on graphene: the proof-of-principle experiments were successful, opening the way to their potential use as lightweight, sensitive components of radiation monitoring systems on Earth or in space. The application of specific graphene/polymer nanocomposites for examining human health parameters was also discussed. Integration of (bio)molecules with the graphene surface was reviewed, and their role was explained, taking into consideration the interactions with the material and how they affect the overall sensing properties of the nanocomposites. One of their most important characteristics is the loading capacity of nanocomposites towards the biological analyte acting as the sensing element. The use of fractal-type nanoparticles with high surface roughness represents a great advantage for the immobilization of large quantities of enzymes or RNA for biosensing purposes.

## Figures and Tables

**Table 1:** Bottom-up techniques for producing graphene sheets. For each technique, the size of graphene sheets that can be obtained and the related advantages/disadvantages are reported.

Technique	Graphene Size		Advantages	Disadvantages	References
	Thickness	Lateral			
Confined self-assembly	Single layer	100 nm	Thickness control	Presence of defects	[62]
Arc discharge	Single layer, bilayer, and few layers	Few 100 nm to few µm	Up to 10 g/h of graphene	Low yield of graphene; carbonaceous impurities	[63]
Epitaxial growth on SiC	Few layers	Up to cm	Very large area of pure graphene	Very small scale	[64,65,66]
CVD	Few layers	Very large (cm)	Large size; high quality	Small production scale	[67]
Reduction of carbon monoxide (CO)	Multiple layers	Sub-μm	Unoxidized sheets	Contamination with α-Al_2_O_3_ and α-Al_2_S	[68]
Unzipping of carbon nanotubes	Multiple layers	Few μm long nanoribbons	Size controlled by selection of the starting nanotubes	Expensive starting material; oxidized graphene	[69]

**Table 2:** Top-down techniques for producing graphene sheets. For each technique, the size of graphene sheets that can be obtained and the related advantages/disadvantages are reported.

Technique	Graphene Size		Advantages	Disadvantages	References
	Thickness	Lateral			
Electrochemical exfoliation/functionalization of graphite	Single and few layers	500–700 nm	High electrical conductivity of the functionalized graphene	Cost of ionic liquids	[70,71]
Direct sonication of graphite	Single and multiple layers	μm or sub-μm	Unmodified graphene; inexpensive	Low yield; separation	[72,73]
Micromechanical exfoliation	Few layers	μm to cm	Large size and unmodified graphene sheets	Very small-scale production	[74]
Superacid dissolution of graphite	Mostly single layer	300–900 nm	Unmodified graphene; scalable	Use of hazardous chlorosulfonic acid; cost of acid removal	[75]
Thermal exfoliation/reduction of graphene oxide	Single and few layer	∼500 nm	1-step exfoliation/reduction; short heating time; dry basis	High heating temperature; smaller sheet size compared to chemically reduced sheets	[76]
Chemical reduction of colloidal graphene oxide in water	Single and multiple layer	μm or sub-μm	Large sheet size; some routes use only water	Some of these methods use hazardous chemicals; only dispersed in hydrophilic polymers	[77]
Li alkylation of graphite fluoride	Single layer	μm	Large size; functionalized sheets; no oxygen functionality	Cost of the starting material; restacking after annealing	[78]

**Table 3:** Main polymers used in the fabrication of graphene-based nanocomposites, with the indication of the fabrication method and the property enhancement induced by the filler.

Polymer Used as Matrix	Type of Graphene Filler	Fabrication Method	Property Enhanced	Reference
Poly(vinyl alcohol) (PVA)	Reduced graphene oxide	Solution blending	Mechanical properties (increase in elastic modulus and tensile strength)	[109]
Poly(methyl methacrylate) (PMMA)	Reduced graphene oxide	Solution blending	Electrical conductivity	[110]
Poly(butylene succinate) (PBS)	Graphene oxide	Solution blending	Mechanical properties (increase in elastic modulus and tensile strength)	[111]
Chitosan	Cryomilled graphene	Solution blending	Mechanical properties (increase in tensile strength)	[112]
Isobutylene isoprene rubber (IIR)	Reduced graphene oxide	Solution blending	Dielectrical permittivity	[113]
Unsaturated polyester resin (UPR)	Graphene nanosheets	Solution blending	Mechanical properties (increase in tensile strength and flexural strength); thermal properties; dielectric strength	[114]
Polystyrene (PS)	Graphene nanosheets	In situ polymerization	Electrical conductivity; thermal properties (increase in glass transition temperature and thermal stability)	[115]
Polyaniline (PANI)	Graphene oxide	In situ polymerization	Electrical conductivity	[116]
High Density Polyethylene (HDPE)	Exfoliated graphene	Melt mixing	Electrical conductivity	[117]
Polycarbonate (PC)	Functionalized graphene sheets	Melt mixing	Electrical conductivity	[118]
Polypropylene (PP)	Exfoliated graphene	Melt mixing	Mechanical properties (increase in flexural strength)	[119]
Poly(vinyl chloride) (PVC)	Graphene nanoplatelets	Melt mixing	Mechanical properties (increase in elastic modulus and tensile strength)	[120]
Polyethylene terephthalate (PET)	Graphene nanosheets	Melt mixing	Electrical conductivity	[121]

## Data Availability

Not applicable.
